# Cerebral White Matter Changes in Young Healthy Individuals With High Trait Anxiety: A Tract-Based Spatial Statistics Study

**DOI:** 10.3389/fneur.2018.00704

**Published:** 2018-08-24

**Authors:** Min Lu, Chunlan Yang, Tongpeng Chu, Shuicai Wu

**Affiliations:** College of Life Science and Bioengineering, Beijing University of Technology, Beijing, China

**Keywords:** trait anxiety, young healthy individuals, diffusion tensor imaging, tract-based spatial statistical analysis, corona radiate, corpus callosum

## Abstract

**Background:** Abnormalities in prespecified and empirical white matter tracts in young patients with anxiety-related disorders have been reported in some diffusion tensor imaging (DTI) studies. However, with few literatures examining the association between the integrity of whole brain white matter and trait anxiety levels in the non-clinical populations, whether white matter changes arise in young healthy individuals with high trait anxiety remains unknown.

**Methods:** We examined whole brain white matter alterations in young healthy individuals with high anxiety but without history of neurological or psychiatric disorders via DTI technology. Group comparison of tract-based spatial statistics (TBSS) was performed to investigate the microstructural diffusion alterations in 38 high anxious subjects in comparison with 34 low anxious subjects matched with age, gender, and degree of education. These analyses controlled for depression to establish specificity to trait anxiety.

**Results:** Young healthy subjects with high trait anxiety had significantly decreased fractional anisotropy (FA) values in multiple clusters, including corona radiate (CR), anterior thalamic radiation (ATR), inferior fronto-occipital fasciculus (IFOF), bilaterally, body, genu, and splenium of corpus callosum (CC) and forceps minor, compared with low trait anxious subjects. For the abnormal FA regions, the other diffusion metrics were also altered slightly.

**Conclusions:** Non-clinical individuals with high anxiety already have white matter alterations in the thalamus-cortical circuit and some emotion-related areas that were widely reported in anxiety-related disorders. The altered white matter may be a vulnerability marker in individuals at high risk of clinical anxiety. These findings can deepen our understanding of the pathological mechanism of anxiety and further support the need for preventive interventions in high anxiety individuals.

## Introduction

Trait anxiety is a personality dimension characterizes by a long-term negative state, represents fear and worrying about future events, and often comes with many functional consequences, such as increased distractibility and attentional bias in favor of threat-related information (1). Appropriate anxiety has a positive effect on facing stress; it can fully mobilize the function of body organs and suitably improve the vigilance and brain response (2). However, individuals may have a high risk of developing numerous anxiety-related disorders, such as generalized anxiety disorder, obsessive-compulsive disorder (OCD), social anxiety disorder, post-traumatic stress disorder (PTSD), and panic disorder when they experience anxiety excessively and regularly (2). Anxiety is not only manifested by patients with clinical disorders but also in normal populations and is generally evaluated by Spielberger's State-Trait Anxiety Inventory (3). Anxiety arises in the context of gradual maturational changes in the brain (4). Thus, improved understanding of the pathophysiology and etiology of young healthy individuals with trait anxiety is expected to have a positive effect on personal and public health.

Previous structural and functional imaging studies in anxiety-related disorders heavily focused on the prespecified pathways [e.g., interplay between the amygdala and prefrontal cortex (PFC)] related to the regulation of emotion (5). Hence, the region of interest (ROI) method is generally applied in magnetic resonance imaging (MRI) studies on anxiety. Functional findings indicated that anxiety levels are negatively correlated with the activity of rostral anterior cingulate cortical and lateral PFC, particularly the processing of task-irrelevant and threat-related stimuli (6). Huggins et al. (7) showed that functional connectivity between the right insular cortex and posterior cingulate cortex is significantly correlated with the trait anxiety of undergraduates. Another volumetric study found a positive correlation of the volumes of the left amygdala and right hippocampus with trait anxiety (8). Meanwhile, white matter tracts can control complex behaviors as the links between the proximal and distal gray matter in different brain regions (9). The above-mentioned functional and morphological varieties further proved the importance of assessing the integrity of white matter tracts in young healthy individuals with trait anxiety.

DTI (10) is a non-invasive imaging technique used in investigating microscopic architecture details of either normal or diseased tissues; it enables the measurement of the diffusion properties of water molecules (11). Previous studies reported the possible relationship between trait anxiety and DTI-derived indices of some specified fiber tracts in subjects with anxiety-related disorders. Lochner et al. (12) demonstrated that patients with OCD have abnormal FA and MD values in the anterior limb of the internal capsule, anterior limb near the head of the caudate, and cingulum when compared with healthy controls. Kim and Whalen (13) showed a negative correlation between trait anxiety scores and mean FA values of the identified amygdala–ventromedial PFC pathway. Moreover, Baur et al. (14) drew a similar conclusion after examining a pooled sample consisting of patients with social anxiety disorder and healthy controls, although they did not observe such correlation among healthy controls. Despite the fact that a large body of literature related to anxiety-related disorders and white matter tracts are available, the relationship of trait anxiety in young healthy persons with the integrity of whole brain white matter is rarely studied. Hence, we hypothesized that cerebral white matter changes have already occurred in young healthy populations with high trait anxiety. Premorbid white matter abnormalities are potential vulnerability markers for trait anxiety and thus can be useful to populations at high risk of anxiety-related disorders.

In our present study, we applied TBSS (15) (https://fsl.fmrib.ox.ac.uk/fsl/fslwiki/TBSS) to investigate the changes in the integrity of the whole brain white matter in young healthy subjects with high trait anxiety scores and those with low trait anxiety scores. The purpose of TBSS are to extract an individual's white matter skeleton and to align and compare the differences among DTI metrics in skeleton of the groups. It can automatically analyze the entire brain white matter skeleton and prevents subjectively brain regions selection. We aimed to assess whether the integrity of the entire brain white matter is altered in young healthy subjects with high trait anxiety.

## Materials and methods

### Participants

A total of 72 righted-handed and healthy undergraduates or postgraduates from the Southwest University Longitudinal Imaging Multimodal (SLIM) Brain Data Repository (China) were selected for the study (http://fcon_1000.projects.nitrc.org/indi/retro/southwestuni_qiu_index.html) (16). All the participants were fluent in Chinese. Clinical information and behavioral variables were obtained, and the Spielberger's State-Trait Anxiety Inventory, the Beck Depression Inventory (BDI) (17), and the combined Raven's matrices test (CRT) (18) were all measured. The CRT can reflect the ability to make a rational judgment and is less affected by individuals' knowledge and education level. On the basis of individual trait anxiety scores, we selected the participants who scored either ≥50 or ≤ 30 (19) and they were divided into two groups, namely the high trait anxiety (HTA) group (ages ranging from 18 to 25) and low trait anxiety (LTA) groups (ages ranging from 18 to 26). The participants conformed to the MRI scanning standard and had no history of neurological or psychiatric disorders or substance abuse according to the self-report questionnaires they answered before the scan. Informed written consent was obtained from each participant prior to the study. The procedures of consent and experiments were approved by the Research Ethics Committee of the Brain Imaging Center of Southwest University and agreed with the standards of the Declaration of Helsinki (1989).

### Procedures

#### Image data acquisition

Diffusion tensor images were collected at the Southwest University Center for Brain Imaging by using a 3.0-T Siemens Trio MRI scanner (Siemens Medical, Erlangen, Germany). The diffusion tensor data for each subject were obtained by using a diffusion-weighted, single-shot spin-echo EPI sequence (repetition time = 11,000 ms, echo time = 98 ms; matrix = 128 × 128; field of view = 256 × 256 mm^2^; voxel size = 2.0 × 2.0 × 2.0 mm^3^; 60 axial slices with 2.0 mm slice thickness), which provided 30 diffusion-encoding gradient directions with a *b*-value of 1,000 s/mm^2^ and a single measurement without a diffusion-weighting gradient (*b* = 0 s/mm^2^). We scanned the subjects three times to increase the signal-to-noise ratio in the scanning sequence. The final NIFTI files had 93 volumes, and the directions and *b*-values can be found in the bval and bvec files.

#### Image analysis

##### Data analysis

All scans were processed by using the PANDA software (20), which is a MATLAB toolbox that integrates FSL (https://fsl.fmrib.ox.ac.uk/fsl/fslwiki), Diffusion Toolkit (http://www.trackvis.org/dtk/), and MRIcron (https://www.nitrc.org/projects/mricron). Then, the following steps were performed: (1) Quality check: images that showed excessive movements were considered damaged and of poor quality and were thus removed. (2) Brain extraction and estimate mask: deletion of non-brain tissues and estimation of brain mask. (3) Crop and eddy current/motion correction: the redundant parts of the images were cropped for memory reduction; images were corrected for the elimination of movements and eddy current-induced distortions. (4) DTI metric calculation: diffusion tensor fitting and diffusion metrics, including FA, MD, AD and RD were calculated with FSL DTIFit. (5) Spatial normalization: the diffusion metrics were normalized spatially to Montreal Neurological Institute (MNI) space by FSL FNIRT.

##### TBSS analysis

First, we non-linearly registered all DTI metrics maps to align them to the target image by selecting predefined target image with the 1 × 1 × 1 mm^3^ resolution in the standard space. Next, all the subjects' DTI metric images in the MNI152 space were merged into a single 4D image containing FA, AD, RD, and MD. Moreover, a mean FA image obtained by averaging all resampled FA images were skeletonized automatically into a group skeleton map. To exclude voxels in the gray matter and cerebrospinal fluid, we thresholded the mean FA skeleton image with a default value of 0.2. Then, a distance map was created. The distance from the individual voxel to the skeleton was used in the projection of DTI metrics values onto the original mean FA skeleton [see the TBSS paper for more detail (15)]. Hence, DTI metric images of all subjects in the WM skeleton were generated and computed in the next statistics step.

#### Statistical analysis

Demographic characteristics and aspects of behavioral data were analyzed with IBM SPSS Statistics 20.0 for Windows. Then, we performed unpaired two-sample *t*-test for age, TAI, SAI, BDI, and CRT scores and χ^2^ tests for gender to investigate whether the two groups significantly differed with regard to self-rating scores (TAI, SAI, BDI, and CRT) and demographic characteristics (gender and age), respectively. The correlations among the behavioral scores were then evaluated by Spearman correlation. A *p*-value of < 0.05 was considered statistical significant.

We performed voxel-wise analyses by using randomization (21), which is a FSL's tool for non-parametric permutation inference on neuroimaging data. It allows modeling and inference through the use of a standard general linear model [GLM (https://fsl.fmrib.ox.ac.uk/fsl/fslwiki/GLM)] design. The effects of trait anxiety on FA, MD, AD, and RD were tested by modeling GLM that allowed BDI to covary. The DTI metrics were calculated by the model, in which the difference between the two groups was adjusted for covariate. The correlations between TAI and BDI scores were significant. We controlled depression by using the BDI scores as covariates in the GLM. A total of 5000 permutations were performed for each contrast. The *p*-value images, which were fully corrected by threshold-free cluster enhancement (TFCE) (22), were thresholded at *p* < 0.05.

## Results

### Demographic characteristics and behavioral data

Group differences in demographic characteristics and behavioral data are shown in Table 1. The groups did not differ significantly with respect to age, gender, and CRT. The HTA group had higher SAI and BDI scores than the LTA group. In the correlation test of behavioral scores, significant correlations among TAI, SAI, and BDI were observed (Table 2).

**Table 1:** Means and SDs of demographic, behavioral data for both anxiety groups.

**Characteristic**	**Group; mean (*****SD*****)**		**Test statistic**	***P*-value**
	**HTA (*N* = 38)**	**LTA (*N* = 34)**		
TAI	54.5 (2.628)	26.177 (2.516)	*t* = 46.58	< 0.001
Age	20.237 (1.3840)	20.471 (2.078)	*t* = −0.567	0.572
Gender (male/female)	19:19	14:20	χ^2^ = 0.563	0.453
SAI	43.026 (9.356)	24.882 (3.883)	*t* = 10.947	< 0.001
BDI	13.421 (7.366)	2.294 (2.877)	*t* = 8.608	< 0.001
CRT	66.263 (4.157)	66.088 (3.108)	*t* = 0.204	0.839

*TAI, Trait Anxiety Inventory; HTA, High Trait Anxiety; LTA, Low Trait Anxiety; SAI, State Anxiety Inventory; BDI, Beck's Depression Inventory; CRT, Combined Ravens Matrices Test*.

**Table 2:** Spearman correlation.

**Variables**	**TAI**	**SAI**	**BDI**	**CRT**
SAI	0.804^*^			
BDI	0.778^*^	0.743^*^		
CRT	0.03	0.226	−0.068	
Age	−0.015	−0.093	0.04	−0.119

* *indicates p < 0.05*.

### TBSS analysis

**Figure 1:**
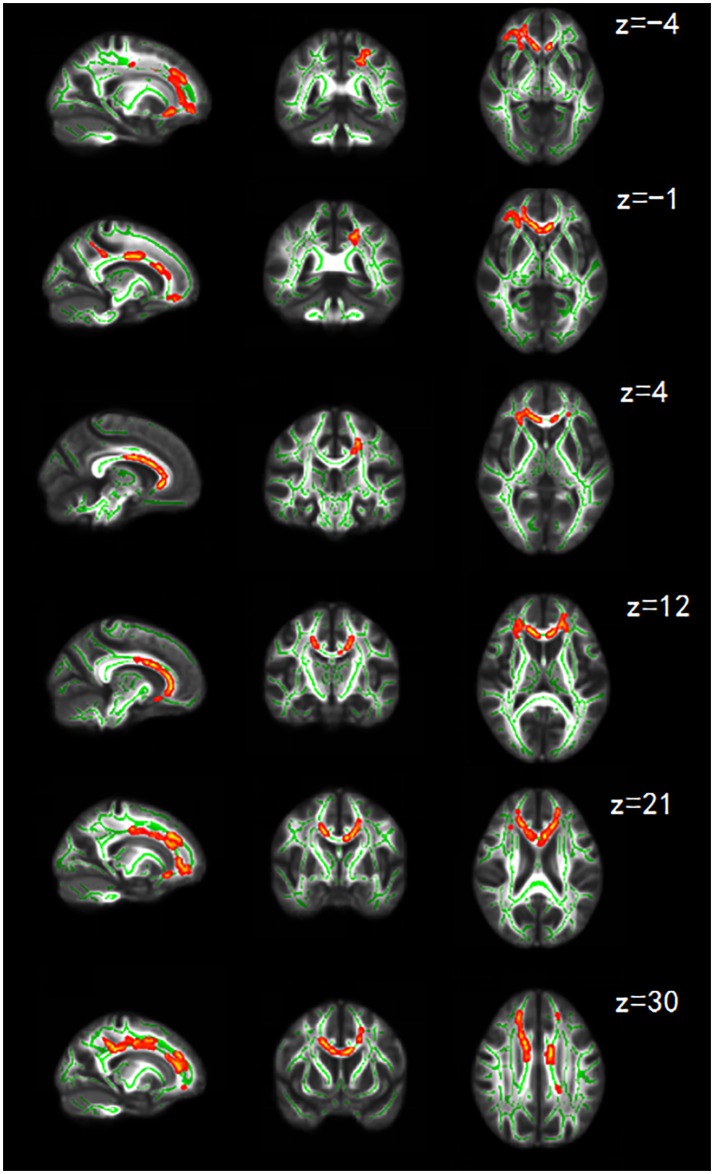
Sagittal coronal and axial maps of FA showing significant differences. FA is lower in the HTA group than that in LTA group in regions including anterior and superior CR bilaterally, left posterior CR, bilateral ATR, bilateral IFOF, body, genu, and splenium of CC and forceps minor. The green color shows the skeleton of the averaged FA and is overlaid on the gray-scale averaged FA map. The red-yellow color shows the clusters of remarkably reduced FA in the HTA group. The threshold for significance was set at *p* < 0.05, which is corrected for TFCE. The right side of the image corresponds to the left hemisphere of the brain. Z represents the coordinates of the axis.

WM abnormalities between the HTA and LTA group mainly involved several WM tracts in the anterior part of the brain. In detail, the HTA group had significantly lower FA values than the LTA group in multiple clusters, including anterior and superior corona radiate (CR) bilaterally, left posterior CR, bilateral anterior thalamic radiation (ATR), bilateral inferior fronto-occipital fasciculus (IFOF), body, genu, and splenium of corpus callosum (CC) and forceps minor. Meanwhile, compared to the LTA group, the HTA group had higher RD and MD values for the above reported abnormal FA clusters. The AD values between the HTA and LTA group were very close. (Figure 1; Table 3).

**Table 3:** Regions of reduced FA in the HTA group compared with the LTA group (*p* < 0.05, TFCE-corrected).

**FA VOXEL-WISE COMPARISON**											
**Cluster voxel**	**Anatomical extent of cluster**	**Mean FA**		**Mean AD (**×**10**^−2^**)**		**Mean RD (**×**10**^−3^**)**		**Mean MD (**×**10**^−3^**)**		**Cluster P**_corr_	**MNI (x, y, z)**
		**HTA**	**LTA**	**HTA**	**LTA**	**HTA**	**LTA**	**HTA**	**LTA**		
4416	Cluster covering parts of:	0.55	0.56	0.12	0.12	0.48	0.46	0.73	0.72	0.032	[12 31 7]
	Body of corpus callosum	0.69	0.72	0.15	0.15	0.38	0.35	0.75	0.74		
	Genu corpus callosum	0.73	0.76	0.15	0.15	0.33	0.31	0.72	0.71		
	Splenium of corpus callosum	0.55	0.58	0.13	0.14	0.51	0.49	0.78	0.78		
	Forceps minor	0.69	0.71	0.14	0.14	0.37	0.35	0.72	0.71		
	Left anterior corona radiate	0.45	0.47	0.11	0.11	0.54	0.52	0.74	0.73		
	Right anterior corona radiate	0.46	0.48	0.11	0.11	0.53	0.51	0.73	0.72		
	Left superior corona radiate	0.55	0.58	0.12	0.12	0.47	0.44	0.72	0.70		
	Right superior corona radiate	0.57	0.59	0.12	0.12	0.44	0.42	0.68	0.68		
	Left posterior corona radiate	0.48	0.51	0.12	0.12	0.55	0.53	0.78	0.77		
	Left anterior thalamic radiation	0.37	0.40	0.11	0.11	0.59	0.57	0.75	0.74		
	Right anterior thalamic radiation	0.37	0.39	0.11	0.11	0.60	0.58	0.75	0.75		
	Left inferior fronto-occipital fasciculus	0.56	0.58	0.13	0.12	0.47	0.45	0.74	0.72		
	Right inferior fronto-occipital fasciculus	0.45	0.49	0.11	0.11	0.53	0.50	0.72	0.72		
47	Cluster covering parts of:	0.59	0.61	0.13	0.13	0.44	0.42	0.74	0.72	0.049	[−16 43 −10]
	Left anterior corona radiate	0.45	0.47	0.11	0.11	0.54	0.52	0.74	0.73		
	Forceps minor	0.69	0.71	0.14	0.14	0.37	0.35	0.72	0.71		

*Clusters where FA value is significantly lower in the HTA group than the LTA group. Mean FA, AD, RD and MD values were reported for the individual regions of the clusters*.

*MNI (x, y, z), coordinates of peak locations in the space of Montreal Neurological Institute (MNI); R, right hemisphere, L, left hemisphere*.

## Discussions

To the best of our knowledge, this work is the first DTI study to examine the integrity of the entire brain white matter and to comprehensively report the FA, AD, RD, and MD values of young healthy subjects with trait anxiety. Overall, the FA values in the CR, ATR, IFOF bilaterally, CC and forceps minor are lower in young healthy subjects with high anxiety than in those with low anxiety. For the reported abnormal regions, other diffusion properties were also changed slightly. These results are more extensive but broader relative to the findings in previous DTI studies on anxiety-related disorders (14, 23–32).

The followings are some considerations on the method. Previous anxiety-related studies based on hypothesis-driven conception generally select some interest or empirical regions to investigate (33–38), and aberrant fibers in reports on anxiety-related disorders are not frequently consistent. The functional connectivity in extensive areas, including auditory, medial visual, and task positive networks, decreases in healthy populations with high anxiety (39). Therefore, we selected a voxel-wise approach to test the alterations in the entire brain white matter in young healthy individuals with high trait anxiety. This approach makes an assumption on the basis of a small portion of a fiber tract (e.g., voxel). Although this sensitive approach cannot eliminate the effects of noise and crossing fibers, thereby resulting in false positive outcomes, it can detect more potential vital voxels and clusters changes associated with young healthy subjects with trait anxiety than other methods.

The CR is the most prominent projection fiber in the brain. The anterior CR (ACR) includes thalamic projections from the internal capsule to the cortex (40, 41). A DTI study on PTSD reported that FA in the right ACR is inversely associated with PTSD severity (31). White matter abnormalities in the ACR can have an effect on the emotional (31) and executive attention network functions (42). In thalamic projections areas, Chiu et al. (24) found decreased FA in the right ATR in OCD. However, increased FA values were found in the right ATR in another OCD study (43). In prior DTI studies on PTSD, the cortical-ACR-IC-thalamus-limbic pathway was suggested to play a key role rather than the more direct cortical-uncinatus fasciculus (UF)-limbic pathway in theory (44–46). Cortical–thalamus–limbic pathway plays a prominent role in emotional behavior and regulation associated with PTSD (47, 48). Similarly, our study showed that alterations in the ACR-thalamus pathway are more serious and extensive than in the UF pathway, manifested as decreased FA values in bilateral CR and ATR regions, in young healthy subjects with high anxiety. These findings, together with the results of the current study, suggest that decreased FA in the ACR-thalamus pathway may be a trait marker in young healthy individuals with high trait anxiety.

The CC, which is the largest white matter structure and commissural fiber in the brain, comprises extensive networks and regulates motor, sensory abilities, attention, intelligence, and emotional states (45, 49). The lack of interhemispheric communication between the hemispheres may cause emotional disturbances (50), and dysfunction in the CC have been implicated in the pathogenesis of some forms of OCD (30, 51). The electrical high-frequency stimulation of the genu of the CC in psychiatric patients immediately eliminates anxiety and tension, and CC pathways may be hyperactive in anxiety and tension (52). The observed FA reduction in the CC may be a vulnerable marker in young healthy subjects with high anxiety.

The IFOF, which integrates the auditory and visual association cortices into the prefrontal cortex, connects the occipital and frontal lobes (53). The IFOF has long been implicated in OCD. Reduced FA is observed in the right IFOF in previous report on adolescents with generalized anxiety disorder (54). Garibotto et al. (26) reported decreased FA along CC, cingulum, SLF, and IFOF bilaterally in OCD. In some studies about PTSD, Kim et al. (55) showed decreased FA in the left anterior cingulum, whereas another small study found increased FA in the same region (56). In a OCD study, increased FA values were found in several white matter tracts, including the major and minor forceps, bilateral corticospinal tract, right ATR and bilateral SLF (43). However, in a PTSD study, there was decreased FA in clusters involving forceps minor (57). Another study found that OCD patients had significantly lower FA, higher MD, and AD values in forceps minor, compared with healthy controls (23). Similar to two studies mentioned above, our research also found decreased FA values in forceps minor. Meanwhile, combined with the discovery of the CC, WM abnormalities on populations with high anxiety involved not only anterior of the CC, but also extending to forceps minor. Therefore, we inferred that pathway of anterior of the CC-forceps minor may also be a vulnerable marker on high anxiety individuals. Taken together, our findings, on young healthy individuals with high anxiety, were intersectant and similar with results in these prior studies on anxiety-related disorders.

These findings should be inferred with caution. Our results suggest that the FA value is more sensitive than any other DTI metrics (MD, AD, RD) in detecting white matter alterations in young healthy samples with high anxiety. The FA reduction was due to an increase in radial diffusivity or a decrease in axial diffusivity, which could be attributed to any or all the following processes: (1) decrease in axonal density (2) decrease in axonal myelination (3) abnormal axonal membranes (4) reorganization of axons at a macroscopic level (58). These factors may cause low directionality and partly decrease FA values in areas associated with high anxiety in young healthy subjects. In our study, we inferred that FA changes is mainly caused by the increase of the radial diffusivity.

In our study, all subjects are college students in late adolescent stage, whose brains were not yet mature. These diffusion changes are diverse in different regions during the developmental period of maturation. A developmental FA increase was found in the ALIC (59–62), IFOF (61, 63, 64), CC (62, 65–68) during the adolescence period. This increase may have affected the brain white matter analysis outcomes. In addition, the FA values in the cerebral white matter follow the inverted U-shaped curves and reach the peak between 20 and 30 years across lifespan (69, 70). Hence, white matter abnormality in high anxiety undergraduates may affect the developmental curve across lifespan. Furthermore, several limits in our current study were identified. First, we only focused on Chinese undergraduates who were right-handed and had no history of psychiatric disorders. Thus, our subjects' data were relatively homogeneous, thereby limiting our findings to inference of specified population. Second, our study lacked multimodal data (e.g., FMRI) to directly correspond with our microstructural results. Although our study had the above-mentioned limitations, we believe that our data provided strong support for white matter abnormalities in young healthy individuals with high anxiety.

In conclusion, our study demonstrated that white matter abnormalities in young healthy individuals with high trait anxiety were mainly involved in or connected to specific regions of the thalamus-cortical circuit and some emotion-related areas that were widely reported in anxiety-related disorders. This preliminary study provides a novel way of detecting vulnerable markers associated with non-clinical trait anxiety. These investigations can elucidate the pathological mechanism of anxiety. The observed cerebral white matter changes in our study further support the need for preventive interventions in high anxiety individuals.

## Ethics statement

This study was carried out in accordance with the recommendations of the Declaration of Helsinki(1989), the Research Ethics Committee of the Brain Imaging Center of Southwest University. The protocol was approved by the Research Ethics Committee of the Brain Imaging Center of Southwest University. All subjects gave written informed consent in accordance with the Declaration of Helsinki.

## Author contributions

ML and CY made contributions to the conception, design, analysis and interpretation of DTI data, and drafted the manuscript. TC made contributions to the data preprocessing. CY and SW made contributions to the revision of the final manuscript. All authors read and approved the final manuscript.

### Conflict of interest statement

The authors declare that the research was conducted in the absence of any commercial or financial relationships that could be construed as a potential conflict of interest.

## Abbreviations

DTI: diffusion tensor imaging
TBSS: tract-based spatial statistics
FA: fractional anisotropy
MD: mean diffusivity
AD: axial diffusivity
RD: radial diffusivity
OCD: obsessive-compulsive disorder
PTSD: post-traumatic stress disorder
PFC: prefrontal cortex
MRI: magnetic resonance imaging
BDI: Beck Depression Inventory
CRT: combined Raven's matrices test
HTA: high trait anxiety
LTA: low trait anxiety
MNI: Montreal Neurological Institute
TFCE: threshold-free cluster enhancement
CR: corona radiate
ACR: anterior CR
CC: corpus callosum
ATR: anterior thalamic radiation
IFOF: inferior fronto-occipital fasciculus
SLF: superior longitudinal fasciculus
UF: unicinate fasciculus.

## References

[1] Modi S Trivedi R Singh K Kumar P Rathore RKS Tripathi RP. Individual differences in trait anxiety are associated with white matter tract integrity in fornix and uncinate fasciculus: preliminary evidence from a DTI based tractography study. Behav Brain Res. (2013) 238:188–92. 10.1016/j.bbr.2012.10.007 23085341

[2] Edition F Diagnostic and Statistical Manual of Mental Disorders. Washington, DC: American Psychiatric Association (2013).

[3] Spielberger CD Gorsuch RL Manual for the State-Trait Anxiety Inventory (form Y):(“Self-Evaluation Questionnaire”). Palo Alto, CA: Consulting Psychologists Press, Incorporated (1983).

[4] Paus T Keshavan M Giedd JN. Why do many psychiatric disorders emerge during adolescence? Nat Rev Neurosci. (2008) 9:947. 10.1038/nrn2513 19002191 PMC2762785

[5] Bishop SJ. Neurocognitive mechanisms of anxiety: an integrative account. Trends Cogn Sci. (2007) 11:307–16. 10.1016/j.tics.2007.05.008 17553730

[6] Bishop S Duncan J Brett M Lawrence AD. Prefrontal cortical function and anxiety: controlling attention to threat-related stimuli. Nat Neurosci. (2004) 7:184–8. 10.1038/nn1173 14703573

[7] Huggins A Belleau E Miskovich T Pedersen W Larson C Altered functional connectivity between right insular cortex and default mode regions associated with perceived stress and anxiety during undergraduate students, finals week. Biol Psychiatry (2017) 81:S328 10.1016/j.biopsych.2017.02.875

[8] Baur V Hänggi J Jäncke L. Volumetric associations between uncinate fasciculus, amygdala, and trait anxiety. BMC Neurosci. (2012) 13:4. 10.1186/1471-2202-13-4 22217209 PMC3398321

[9] Fields RD. White matter in learning, cognition and psychiatric disorders. Trends Neurosci. (2008) 31:361–70. 10.1016/j.tins.2008.04.001 18538868 PMC2486416

[10] Basser PJ Mattiello J LeBihan D. MR diffusion tensor spectroscopy and imaging. Biophys J. (1994) 66:259–67. 10.1016/S0006-3495(94)80775-1 8130344 PMC1275686

[11] Mori S Zhang J. Principles of diffusion tensor imaging and its applications to basic neuroscience research. Neuron (2006) 51:527–39. 10.1016/j.neuron.2006.08.012 16950152

[12] Lochner C Fouché JP du Plessis S Spottiswoode B Seedat S Fineberg N. Evidence for fractional anisotropy and mean diffusivity white matter abnormalities in the internal capsule and cingulum in patients with obsessive–compulsive disorder. J Psychiatry Neurosci. (2012) 37:193–9. 10.1503/jpn.110059 22297066 PMC3341411

[13] Kim MJ Whalen PJ. The structural integrity of an amygdala-prefrontal pathway predicts trait anxiety. J Neurosci. (2009) 29:11614–8. 10.1523/JNEUROSCI.2335-09.2009 19759308 PMC2791525

[14] Baur V Hänggi J Rufer M Delsignore A Jäncke L Herwig U. White matter alterations in social anxiety disorder. J Psychiatric Res. (2011) 45:1366–72. 10.1016/j.jpsychires.2011.05.007 21705018

[15] Smith SM Jenkinson M Johansen-Berg H Rueckert D Nichols TE Mackay CE. Tract-based spatial statistics: voxelwise analysis of multi-subject diffusion data. Neuroimage (2006) 31:1487–505. 10.1016/j.neuroimage.2006.02.024 16624579

[16] Liu W Wei D Chen Q Yang W Meng J Wu G. Longitudinal test-retest neuroimaging data from healthy young adults in southwest China. Sci Data (2017) 4:170017. 10.1038/sdata.2017.17 28195583 PMC5308199

[17] Beck AT Ward CH Mendelson M Mock J Erbaugh J. An inventory for measuring depression. Arch Gen Psychiatry (1961) 4:561–71. 10.1001/archpsyc.1961.01710120031004 13688369

[18] Li D Hu KD Chen GP Jin Y Li M The testing results report on the combined Raven's test in Shanghai. Psychol Sci. (1988) 4:27–31.

[19] Eden AS Schreiber J Anwander A Keuper K Laeger I Zwanzger P. Emotion regulation and trait anxiety are predicted by the microstructure of fibers between amygdala and prefrontal cortex. J Neurosci. (2015) 35:6020–7. 10.1523/JNEUROSCI.3659-14.2015 25878275 PMC6605169

[20] Cui Z Zhong S Xu P Gong G He Y. PANDA: a pipeline toolbox for analyzing brain diffusion images. Front Hum Neurosci. (2013) 7:42. 10.3389/fnhum.2013.00042 23439846 PMC3578208

[21] Winkler AM Ridgway GR Webster MA Smith SM Nichols TE. Permutation inference for the general linear model. NeuroImage (2014) 92:381–97. 10.1016/j.neuroimage.2014.01.060 24530839 PMC4010955

[22] Smith SM Nichols TE. Threshold-free cluster enhancement: addressing problems of smoothing, threshold dependence and localisation in cluster inference. Neuroimage (2009) 44:83–98. 10.1016/j.neuroimage.2008.03.061 18501637

[23] Benedetti F Giacosa C Radaelli D Poletti S Pozzi E Dallaspezia S. Widespread changes of white matter microstructure in obsessive–compulsive disorder: effect of drug status. Eur Neuropsychopharmacol. (2013) 23:581–93. 10.1016/j.euroneuro.2012.07.002 22954900

[24] Chiu C-H Lo Y-C Tang H-S Liu I-C Chiang W-Y Yeh F-C. White matter abnormalities of fronto-striato-thalamic circuitry in obsessive–compulsive disorder: a study using diffusion spectrum imaging tractography. Psychiatry Res. (2011) 192:176–82. 10.1016/j.pscychresns.2010.09.009 21546223

[25] Fontenelle L Bramati I Moll J Mendlowicz M de Oliveira R Tovar-Moll F. White matter changes in ocd revealed by diffusion tensor imaging. CNS Spectrums (2011) 16:101–9. 10.1017/S1092852912000260 24725386

[26] Garibotto V Scifo P Gorini A Alonso CR Brambati S Bellodi L. Disorganization of anatomical connectivity in obsessive compulsive disorder: a multi-parameter diffusion tensor imaging study in a subpopulation of patients. Neurobiol Disease (2010) 37:468–76. 10.1016/j.nbd.2009.11.003 19913616

[27] Jackowski AP Douglas-Palumberi H Jackowski M Win L Schultz RT Staib LW. Corpus callosum in maltreated children with posttraumatic stress disorder: a diffusion tensor imaging study. Psychiatry Res. (2008) 162:256–61. 10.1016/j.pscychresns.2007.08.006 18296031 PMC3771642

[28] Liao W Xu Q Mantini D Ding J Machado-de-Sousa J Hallak EC. Altered gray matter morphometry and resting-state functional and structural connectivity in social anxiety disorder. Brain Res. (2011) 1388:167–77. 10.1016/j.brainres.2011.03.018 21402057

[29] Nakamae T Narumoto J Sakai Y Nishida S Yamada K Nishimura T. Diffusion tensor imaging and tract-based spatial statistics in obsessive-compulsive disorder. J Psychiatr Res. (2011) 45:687–90. 10.1016/j.jpsychires.2010.09.016 20965515

[30] Saito Y Nobuhara K Okugawa G Takase K Sugimoto T Horiuchi M. Corpus callosum in patients with obsessive-compulsive disorder: diffusion-tensor imaging study. Radiology (2008) 246:536–42. 10.1148/radiol.2462061469 18180336

[31] Sanjuan PM Thoma R Claus ED Mays N Caprihan A. Reduced white matter integrity in the cingulum and anterior corona radiata in posttraumatic stress disorder in male combat veterans: a diffusion tensor imaging study. Psychiatry Res. (2013) 214:260–8. 10.1016/j.pscychresns.2013.09.002 24074963 PMC3988979

[32] Tupler LA Davidson JR Smith RD Lazeyras F Charles HC Krishnan KR. A repeat proton magnetic resonance spectroscopy study in social phobia. Biol Psychiatry (1997) 42:419–24. 10.1016/S0006-3223(96)00501-X 9285077

[33] Adhikari A Topiwala MA Gordon JA. Synchronized activity between the ventral hippocampus and the medial prefrontal cortex during anxiety. Neuron (2010) 65:257–69. 10.1016/j.neuron.2009.12.002 20152131 PMC2822726

[34] Blackmon K Barr WB Carlson C Devinsky O DuBois J Pogash D. Structural evidence for involvement of a left amygdala-orbitofrontal network in subclinical anxiety. Psychiatry Res. (2011) 194:296–303. 10.1016/j.pscychresns.2011.05.007 21803551 PMC3544472

[35] Coutinho JF Fernandesl SV Soares JM Maia L Gonçalves ÓF Sampaio A. Default mode network dissociation in depressive and anxiety states. Brain Imaging Behav. (2016) 10:147–57. 10.1007/s11682-015-9375-7 25804311

[36] Kazlouski D Rollin MDH Tregellas J Shott ME Jappe LM Hagman JO. Altered fimbria-fornix white matter integrity in anorexia nervosa predicts harm avoidance. Psychiatry Res. (2011) 192:109–16. 10.1016/j.pscychresns.2010.12.006 21498054 PMC3085716

[37] Rauch SL Shin LM Wright CI. Neuroimaging studies of amygdala function in anxiety disorders. Ann N Y Acad Sci. (2006) 985:389–410. 10.1111/j.1749-6632.2003.tb07096.x 12724173

[38] Tao Y Liu B Zhang X Li J Qin W Yu C. The structural connectivity pattern of the default mode network and its association with memory and anxiety. Front Neuroanatomy (2015) 9:152. 10.3389/fnana.2015.00152 26635544 PMC4659898

[39] Modi S Kumar M Kumar P Khushu S. Aberrant functional connectivity of resting state networks associated with trait anxiety. Psychiatry Res. (2015) 234:25–34. 10.1016/j.pscychresns.2015.07.006 26385540

[40] Catani M Howard RJ Pajevic S Jones DK. Virtual *in vivo* interactive dissection of white matter fasciculi in the human brain. NeuroImage (2002) 17:77–94. 10.1006/nimg.2002.1136 12482069

[41] Wakana S Jiang H Nagae-Poetscher LM van Zijl PCM Mori S. Fiber tract–based atlas of human white matter anatomy. Radiology (2004) 230:77–87. 10.1148/radiol.2301021640 14645885

[42] Yin X Han Y Ge H Xu W Huang R Zhang D. Inferior frontal white matter asymmetry correlates with executive control of attention. Hum Brain Mapp. (2013) 34:796–813. 10.1002/hbm.21477 22110013 PMC6869851

[43] Zarei M Mataix-Cols D Heyman I Hough M Doherty J Burge L. Changes in gray matter volume and white matter microstructure in adolescents with obsessive-compulsive disorder. Biol Psychiatry (2011) 70:1083–90. 10.1016/j.biopsych.2011.06.032 21903200

[44] Ayling E Aghajani M Fouche J-P van der Wee N. Diffusion tensor imaging in anxiety disorders. CurrPsychiatry Rep. (2012) 14:197–202. 10.1007/s11920-012-0273-z 22460663

[45] Gazzaniga MS. Cerebral specialization and interhemispheric communicationDoes the corpus callosum enable the human condition? Brain (2000) 123:1293–326. 10.1093/brain/123.7.1293 10869045

[46] Von Der Heide RJ Skipper LM Klobusicky E Olson IR. Dissecting the uncinate fasciculus: disorders, controversies and a hypothesis. Brain (2013) 136:1692–707. 10.1093/brain/awt094 23649697 PMC3673595

[47] Drevets WC Price JL Furey ML. Brain structural and functional abnormalities in mood disorders: implications for neurocircuitry models of depression. Brain Struct Funct. (2008) 213:93–118. 10.1007/s00429-008-0189-x 18704495 PMC2522333

[48] Schuff N Zhang Y Zhan W Lenoci M Ching C Boreta L. Patterns of altered cortical perfusion and diminished subcortical integrity in posttraumatic stress disorder: an MRI study. Neuroimage (2011) 54 (Suppl. 1):S62. 10.1016/j.neuroimage.2010.05.024 20483375 PMC2945438

[49] Tamietto M Adenzato M Geminiani G de Gelder B. Fast recognition of social emotions takes the whole brain: interhemispheric cooperation in the absence of cerebral asymmetry. Neuropsychologia (2007) 45:836–43. 10.1016/j.neuropsychologia.2006.08.012 16996092

[50] Paul LK Lautzenhiser A Brown WS Hart A Neumann D Spezio M. Emotional arousal in agenesis of the corpus callosum. Int J Psychophysiol. (2006) 61:47–56. 10.1016/j.ijpsycho.2005.10.017 16759726

[51] Farchione TR Lorch E Rosenberg DR. Hypoplasia of the corpus callosum and obsessive-compulsive symptoms. J Child Neurol. (2002) 17:535–7. 10.1177/088307380201700712 12269734

[52] Laitinen L Stereotactic lesions in the knee of the corpus callosum in the treatment of emotional disorders. Lancet (1972) 299:472–5. 10.1016/S0140-6736(72)90124-9 4109819

[53] Catani M. Diffusion tensor magnetic resonance imaging tractography in cognitive disorders. Curr Opin Neurol. (2006) 19:599–606. 10.1097/01.wco.0000247610.44106.3f 17102700

[54] Liao M Yang F Zhang Y He Z Su L Li L. White matter abnormalities in adolescents with generalized anxiety disorder: a diffusion tensor imaging study. BMC Psychiatry (2014) 14:41. 10.1186/1471-244X-14-41 24528558 PMC3937009

[55] Kim SJ Jeong D-U Sim ME Bae SC Chung A Kim MJ. Asymmetrically altered integrity of cingulum bundle in posttraumatic stress disorder. Neuropsychobiology (2006) 54:120–5. 10.1159/000098262 17199097

[56] Abe O Yamasue H Kasai K Yamada H Aoki S Iwanami A. Voxel-based diffusion tensor analysis reveals aberrant anterior cingulum integrity in posttraumatic stress disorder due to terrorism. Psychiatry Res. (2006) 146:231–42. 10.1016/j.pscychresns.2006.01.004 16545552

[57] Olson EA Cui J Fukunaga R Nickerson LD Rauch SL Rosso IM. Disruption of white matter structural integrity and connectivity in posttraumatic stress disorder: a TBSS and tractography study. Depress Anxiety (2017) 34:437–45. 10.1002/da.22615 28294462 PMC5407943

[58] Simmonds DJ Hallquist MN Asato M Luna B. Developmental stages and sex differences of white matter and behavioral development through adolescence: a longitudinal diffusion tensor imaging (DTI) study. NeuroImage (2014) 92:356–68. 10.1016/j.neuroimage.2013.12.044 24384150 PMC4301413

[59] Bonekamp D Nagae LM Degaonkar M Matson M Abdalla WM Barker PB. Diffusion tensor imaging in children and adolescents: reproducibility, hemispheric, and age-related differences. Neuroimage (2007) 34:733–42. 10.1016/j.neuroimage.2006.09.020 17092743 PMC1815474

[60] Giorgio A Watkins KE Chadwick M James S Winmill L Douaud G. Longitudinal changes in grey and white matter during adolescence. NeuroImage (2010) 49:94–103. 10.1016/j.neuroimage.2009.08.003 19679191

[61] Schmithorst VJ Wilke M Dardzinski BJ Holland SK. Correlation of white matter diffusivity and anisotropy with age during childhood and adolescence: a cross-sectional diffusion-tensor MR imaging study. Radiology (2002) 222:212–8. 10.1148/radiol.2221010626 11756728 PMC2268734

[62] Snook L Paulson L-A Roy D Phillips L Beaulieu C. Diffusion tensor imaging of neurodevelopment in children and young adults. Neuroimage (2005) 26:1164–73. 10.1016/j.neuroimage.2005.03.016 15961051

[63] Eluvathingal TJ Hasan KM Kramer L Fletcher JM Ewing-Cobbs L. Quantitative diffusion tensor tractography of association and projection fibers in normally developing children and adolescents. Cerebral Cortex (2007) 17:2760–8. 10.1093/cercor/bhm003 17307759 PMC2084482

[64] Lebel C Walker L Leemans A Phillips L Beaulieu C. Microstructural maturation of the human brain from childhood to adulthood. NeuroImage (2008) 40:1044–55. 10.1016/j.neuroimage.2007.12.053 18295509

[65] Ashtari M Cervellione KL Hasan KM Wu J McIlree C Kester H. White matter development during late adolescence in healthy males: a cross-sectional diffusion tensor imaging study. Neuroimage (2007) 35:501–10. 10.1016/j.neuroimage.2006.10.047 17258911

[66] Barnea-Goraly N Menon V Eckert M Tamm L Bammer R Karchemskiy A. White matter development during childhood and adolescence: a cross-sectional diffusion tensor imaging study. Cerebral Cortex (2005) 15:1848–54. 10.1093/cercor/bhi062 15758200

[67] Giorgio A Watkins KE Douaud G James AC James S De Stefano N. Changes in white matter microstructure during adolescence. Neuroimage (2008) 39:52–61. 10.1016/j.neuroimage.2007.07.043 17919933

[68] Muetzel RL Collins PF Mueller BA Schissel AM Lim KO Luciana M. The development of corpus callosum microstructure and associations with bimanual task performance in healthy adolescents. Neuroimage (2008) 39:1918–25. 10.1016/j.neuroimage.2007.10.018 18060810 PMC2408381

[69] Hasan KM Kamali A Iftikhar A Kramer LA Papanicolaou AC Fletcher JM. Diffusion tensor tractography quantification of the human corpus callosum fiber pathways across the lifespan. Brain Research (2009) 1249:91–100. 10.1016/j.brainres.2008.10.026 18996095 PMC3727909

[70] Kochunov P Williamson DE Lancaster J Fox P Cornell J Blangero J. Fractional anisotropy of water diffusion in cerebral white matter across the lifespan. Neurobiol Aging (2012) 33:9–20. 10.1016/j.neurobiolaging.2010.01.014 20122755 PMC2906767

